# In Vitro Investigations of Human Bioaccessibility from Reference Materials Using Simulated Lung Fluids

**DOI:** 10.3390/ijerph14020112

**Published:** 2017-01-24

**Authors:** Aurélie Pelfrêne, Mark R. Cave, Joanna Wragg, Francis Douay

**Affiliations:** 1 Laboratoire Génie Civil et géo-Environnement (LGCgE), ISA Lille, Yncréa Hauts-de-France, 48 Boulevard Vauban, Lille Cedex 59046, France; francis.douay@yncrea.fr; 2 British Geological Survey, Keyworth, Nottingham NG12 5GG, UK; mrca@bgs.ac.uk (M.R.C.); jwrag@bgs.ac.uk (J.W.)

**Keywords:** airborne particulate matter, metallic elements, bioaccessibility, simulated lung fluids

## Abstract

An investigation for assessing pulmonary bioaccessibility of metals from reference materials is presented using simulated lung fluids. The objective of this paper was to contribute to an enhanced understanding of airborne particulate matter and its toxic potential following inhalation. A large set of metallic elements (Ba, Cd, Co, Cr, Cu, Mn, Ni, Pb, Sr, and Zn) was investigated using three lung fluids (phosphate-buffered saline, Gamble’s solution and artificial lysosomal fluid) on three standard reference materials representing different types of particle sources. Composition of the leaching solution and four solid-to-liquid (S/L) ratios were tested. The results showed that bioaccessibility was speciation- (i.e., distribution) and element-dependent, with percentages varying from 0.04% for Pb to 86.0% for Cd. The higher extraction of metallic elements was obtained with the artificial lysosomal fluid, in which a relative stability of bioaccessibility was observed in a large range of S/L ratios from 1/1000 to 1/10,000. For further investigations, it is suggested that this method be used to assess lung bioaccessibility of metals from smelter-impacted dusts.

## 1. Introduction

Because a wide variety of natural and anthropogenic materials and chemicals are recognized to influence human health, a great deal of research has been focused on the determination of harmful elements in airborne particulate matter (APM). The main earth materials linked to the human health include: (1) mineral dusts of asbestos and some other asbestiform or fibrous minerals, crystalline silica and coal generated by the natural weathering of rocks and by anthropogenic activities (such as mining, industrial, construction and demolition activities); (2) solid, gaseous and aqueous wastes or byproducts of mining, mineral processing, smelting and energy production; (3) construction materials such as cement, concrete, aggregate, mineral and glass fiber insulation, and gypsum wallboard; (4) soils and dusts containing metals, organic contaminants or pathogens; (5) volcanic ashes and gases [1]. A variety of diseases or health problems is associated with exposure to elements found in earth materials (e.g., asbestosis, silicosis, lung cancer, industrial bronchitis, “black lung”, fibrosis, irritation of respiratory tract, asthma; [2,3,4]) and depends on their solubility, i.e., degree to which the materials are solubilized in body fluids [5]. The elements from earth materials include (1) essential/beneficial elements (such as Ca, Co, Cu, Cr, F, I, Fe, Mg, Mo, Mn, Se, and Zn) for which health problems may result from either deficiencies or excesses, and (2) nonessential elements (such as Al, As, Be, Cd, Pb, Hg, Ni, radionuclides) which are toxic in excess [1,6]. Occupational as well as environmental exposure to these elements concerns workers and populations living in urban and industrial areas. The first studies consisted of determining concentrations and composition of APM at different urban and industrial sites and work places. In the industrial sector, the dusts have been intensively investigated from the viewpoint of their effects on human health (e.g., [7,8,9,10,11,12]). Human exposure to particulate matters, originating from vehicular traffic, road dust resuspension, dust storm, long-range transport, combustion and fumes in daily environment, has recently been of increasing interest despite being an issue of importance over the past few decades (e.g., [13,14,15,16,17,18,19]). Indeed, particulate matter is a real problem for the public health of the population in urban areas since epidemiological studies have shown that they are associated with the increase of respiratory and cardiovascular mortality and morbidity [20,21,22,23].

Most of the studies dealing with determination of metals/metalloids in APM focus on the measurement of total concentrations without distinguishing the various species that are present [24]. This commonly-applied determination provides an upper-end estimate of potential metal toxicity. For improved risk assessment, it is important to consider the solubility of metallic elements present in inhaled APM, addressed by the term lung bioaccessibility, which is defined as the amount of a substance soluble in a simulated lung fluid environment and capable of inducing pulmonary toxicity [25,26,27,28]. Therefore, it is necessary to study the impact of easily released metallic components in APM on human health.

Over the last thirty years, numerous studies have been carried out to develop in vitro respiratory uptake tests to measure the short-term solubility of elements in earth materials. Tests were developed to simulate the effects of lung fluids on bioaccessibility and used as an alternative to in vivo assays in health studies [29]. These studies vary considerably in their physical design; particle size, shape, and other characteristics of the material tested such as fluid composition, test duration, and other parameters [30,31]. The fate of inhaled materials in the respiratory tract is a function of their solubility in the fluids lining the lungs, i.e., the extracellular lung fluids and the fluids in the alveolar macrophage. The extracellular lung fluids form a complex and dynamic mixture of several components [32,33,34]: (1) surfactants that include more than 90% lipids (phospholipids), with the remaining 10% composed of a mixture of proteins; (2) mucus material that is composed primarily of glycomucoproteins; and (3) an aqueous serum transudate. In the alveolar macrophages, lysosomal fluids are characterized in terms of their high concentrations of proteases, bactericidal enzymes (such as lysozyme), and other cytotoxic chemicals [35].

Various analytical procedures have been used for the measurement of bioaccessible metal fractions in APM, and were compiled in recent reviews [36,37]. To resume, a variety of dissolution media ranging from simple leaching solutions to simulated lung fluids (SLFs) has been designed to study the fate of inhaled substances. Some studies employ water extraction because of analytical constraints generated by using complex solutions simulating the pulmonary surfactant (e.g., [38,39,40,41,42,43]). However, the ionic strength of the lung fluid differs strongly from that of water. Similarly, sequential extractions have been also performed [17,44,45]. These procedures include different fractions such as water-soluble, ion-exchangeable, carbonate, reducible, organic carbon-associated, residual or alumino-silicate-associated fractions. However, these procedures have been developed specifically for soil and include steps employing strong acid reagents that are not representative of the lung pH conditions. Other more complex solutions have been used to tentatively reproduce the composition of lung fluids: (1) phosphate-buffered saline (PBS; [46,47,48,49,50,51,52]), a standard physiological solution that mimics the ion strength of human blood; (2) Gamble’s solution, that mimics the interstitial fluid within the deep lung, i.e., the surfactant fluids released by type II alveolar cells [34,49,53,54,55]; and (3) artificial lysosomal fluid (ALF) that simulates intercellular conditions in lung cells occurring in conjunction with phagocytosis, therefore representing relatively harsh condition [10,48,49,55,56]. Other solvents have been used more rarely in the literature for the dissolution test, e.g., Eagle’s basal medium [57,58,59], Hank’s buffered salt solution [60,61], and pyrogallol [53].

Various leaching agents have been reported in the literature for determination of bioaccessible metal fractions present within APM. Over the last decade, a limited number of studies have concentrated on the development of in vitro methods to estimate lung bioaccessibility of metals [16,48,62,63,64]. However, no methodology has yet emerged as the ideal choice and none of the existing protocols has been validated via systemic comparison with in vivo data. To contribute to an enhanced understanding of APM and its toxic potential following inhalation, the present study aims to assess the bioaccessibility of metals (Ba, Cd, Co, Cr, Cu, Mn, Ni, Pb, Sr, and Zn, some of them being known as potentially toxic and/or largely present in APM) from three standard reference materials (BCR-723, NIST 2710a and NIST 1648a). To achieve this, the bioaccessible metal fractions were determined: (1) using three SLFs (PBS, Gamble’s solution and ALF) and (2) testing four solid-to-liquid (S/L) ratios (from 1/1000 to 1/10,000). The interest of this work was to define the most appropriate protocol for the assessment, in further investigations, of lung bioaccessibility of metals from smelter-impacted dusts.

## 2. Materials and Methods

### 2.1. Reference Materials

Three standard reference materials (SRMs) were selected to assess bioaccessibility of metals for the inhalation exposure route: BCR-723 (road dust collected in Austria with a particle size fraction of <90 µm and a median value of 14.6 µm), NIST 2710a (soil particles collected in the USA with a particle size fraction of <74 µm), and NIST 1648a (urban atmospheric particulate matter collected in the city of St Louis, MO, USA with a particle size fraction of <100 µm and a median value of 5.85 µm). These samples represent different types of particle sources with a large array of total elemental concentrations (Table 1).

### 2.2. In Vitro Inhalation Bioaccessibility Protocol

Based on the literature, different dissolution media (PBS, Gamble’s solution, modified Gamble’s solution and ALF) were used to estimate the bioaccessibility of metals in the three SRMs. PBS is a standard physiological solution for general use in biological research and medical health care. It mimics the ion strength and pH of human blood and the solution is simple in composition and stable in use, making it suitable for tests of methodological parameters. Gamble’s solution and ALF are complex artificial biological media that simulate different interstitial conditions in the lung. The former corresponds to the interstitial fluid deep within the lung in normal health conditions, whereas the latter simulates conditions occurring in conjunction to phagocytosis by cells, i.e., similar to an immunological reaction of the body [49]. Many studies used Gamble’s solution or slightly modified versions. Modifications included adjusting the pH, addition of surfactant, and not performing the test in anoxic conditions with argon gas (e.g., [15,19,53,62,66,67]). In the present study, Gamble’s solution and modified Gamble’s solution with addition of 0.01% (w/v) dipalmitoyl phosphatidyl choline (DPPC) were tested in order to investigate the influence of this surfactant on the dissolution profile of metals (Table 2). Solutions were freshly prepared prior to extraction.

This experiment was conducted to study the influence of and to estimate the impact of the S/L ratio on pulmonary bioaccessibility of metals in the SRMs. The values of S/L ratio (expressed as g·mL^−1^) were 1/1000, 1/2000, 1/5000 and 1/10,000. In each case, 10 mg of sample was weighed and then 10, 20, 50 and 100 mL, respectively, of dissolution medium was added. The pH of the fluids (7.3 ± 0.1 for PBS, Gamble’s solution and modified Gamble’s solution, and 4.5 ± 0.1 for ALF; Table 2) was adjusted if necessary with HCl or NaOH. The tubes were incubated using an end-over-end rotation in an oven at 37 °C and the tests were not performed in anoxic conditions. After a fixed extraction-time of 24 h, separation of particles from the solutions was performed by centrifugation at 4500 g for 15 min. All experiments were performed with a fixed extraction time of 24 h. Several studies showed that this time is relevant to estimate the upper limit of metal bioaccessibility in APM [48,49,62,68]. Moreover, this time was also considered to take into account a reasonably time-consuming in vitro procedure. The resulting supernatants were removed, filtered through a 0.45 µm filter and stored at <4 °C until analysis. All experiments were performed in triplicate. The bioaccessible metal concentrations in the lung extraction fluids were determined using a Perkin Elmer Optima 7300DV Inductively Coupled Plasma Optical Emission Spectroscopy (ICP-OES) (Perkin Elmer Inc., Waltham, MA, USA). Blank extractions were carried out within each extraction run in order to check for the potential contamination from the reaction vessels and the reagents. For the ICP-OES determination of metals, all calibration and quality control standards were matched by using several metal standards covering the concentration range of interest. The instrument was re-calibrated after not more than 20 samples. Quality control standards (high and low range) were analyzed after testing of no more than 10 unknown samples and at the end of the analytical run.

For each metal, the bioaccessibility is expressed as a ratio between the extracted concentrations in the SLFs and the total concentrations certified in the SRMs.

### 2.3. Statistical Analyses

Statistical analysis of the data was performed using XLSTAT 2013-5-09 (Addinsoft, New York, NY, USA). All data distributions were checked for normality and homogeneity of variances. One-way analysis of variance (ANOVA) was carried out for data that met the conditions of normal distribution and homogeneous variances, and the Tukey test was used for multiple mean comparisons. Data with a distribution deviating from normality were analyzed with the Kruskal-Wallis test. A probability level of *p* < 0.05 was chosen to establish statistical significance.

## 3. Results and Discussion

### 3.1. Influence of the S/L Ratio on Bioaccessibility

The quantity of inhaled materials reaching the alveolar region can greatly vary as a function of human beings (individual physiology), size and concentration of loading particles. The volume of lung fluid may also fluctuate. Rennard et al. [69] estimated that the total lung surface fluid volume in humans ranges from about 15–70 mL. In the literature, many studies estimate that the volume in alveolar regions is relatively small. The volume of extracellular fluid has been estimated to be about 4–9 mL in humans by Anderson [70]. For alveolar fluid, Weibel’s average thickness of 0.068 µm multiplied by an average human alveolar surface area of 100 m^2^ gave an average volume of about 7 mL [71]. Macklin [72] used an average alveolar fluid depth of 0.2 µm covering a surface area of 100 m^2^ to give a total alveolar fluid volume of 20 mL. It is thus difficult to specify a precise S/L ratio value relative to the human physiology. Considering conditions of inhalation exposure for 24 h under a large range of particle concentrations from 20 µg·m^−3^ to 500 µg·m^−3^, and assuming that 100% of the inhaled particles reach the pulmonary alveoli with a daily air uptake between 10 and 20 m^3^ and a total alveolar fluid volume ranging from 5 to 20 mL, the S/L ratio should vary between 1/100,000 and 1/500 (g·mL^−1^). The published papers provided S/L ratio values ranging from 1/20,000 to 1/2 (g·mL^−1^). Several studies have demonstrated that the S/L ratio could influence material dissolution. Midlander et al. [48] showed that for S/L ratios ranging between 1/50, 1/500 and 1/5000 (g·mL^−1^) the metal release rates in dissolution medium (PBS) decreased with increasing particle loading, and more particularly for the highest particle loading of 1/50. In another study, the same authors highlighted no differences between 1/10,000 and 1/5000 in the same dissolution medium [49]. In Caboche et al. [62], different S/L ratios ranging from 1/50,000 to 1/30 were tested in Gamble’s solution. These authors showed a relative stability of metal bioaccessibility between 1/50,000 and 1/500; S/L ratios above 1/500 present a risk of saturation of the solution or competition between the soluble elements. Moreover, a larger mass loading may limit the exposition of the particle surface to the dissolution media.

**Figure 1:**
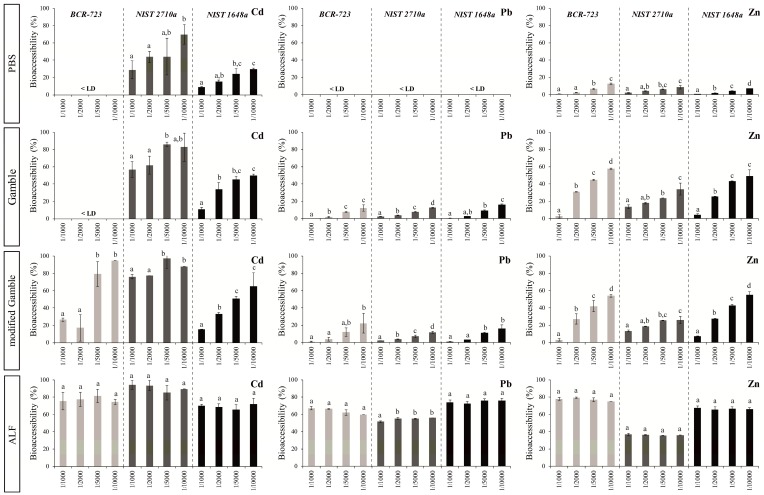
Bioaccessibility values (%; mean and SD; *n* = 3) of Cd, Pb and Zn in the three reference materials obtained through the four lung fluid extractions according to the four S/L ratios. Lowercase letters (a–d) denote significant differences (*p* < 0.05) between the four solid-to-liquid (S/L) ratios for each reference material. LD: limit of detection.

Taking into account the results of the literature and the experimental/analytical constraints, four ratios ranging from 1/1000 to 1/10,000 were used to assess their influence on bioaccessibility of metallic elements in the three SRMs and in the four lung fluids studied. Because similar trends were observed for the ten elements (See Supplementary Materials; Tables S1–S3), Figure 1 presents the results for Cd, Pb and Zn. The results showed an impact of S/L ratio on bioaccessibility of metals in PBS and Gamble’s solutions. In these lung fluids, the metal release increased with decreasing particle loading. A reduction of bioaccessible percentages was observed, ranging from 6.4 to 97.6% for the highest S/L ratio depending on the element and the SRM. However, the metal bioaccessibility was stable in ALF. Apart from such extreme circumstances, the bioaccessibility of metallic elements is independent of S/L ratios, in a range varying from 1/1000 to 1/10,000 when an ALF solution is used.

### 3.2. Comparison of in vitro Tests Using PBS, Gamble’s Solution and ALF

Table 3 presents the bioaccessibility values of ten metallic elements in the three reference materials for the S/L ratio of 1/5000 in the three SLFs studied (PBS, Gamble’s solution and ALF). Results are presented in terms of percent of available metals that have been solubilized to allow comparison between extraction fluids and between elements.

A high bioaccessibility in ALF was evidenced for Ba, Cd, Cu, Mn, Ni, Pb and Zn in the three SRMs, whereas the results showed considerably lower metal release from samples exposed to PBS. These results demonstrated that ALF can dissolve more compounds than PBS. In Gamble’s solution, the elements presented an intermediate behavior. More specifically, total release of these elements decreased according to the following sequence: ALF > Gamble’s solution > PBS. For example: (1) the bioaccessibility of Cd measured in the NIST 1648a was on average 24.1%, 45.2% and 65.6% for PBS, Gamble’s solution and ALF, respectively, (2) for Cu, the bioaccessible values in the BCR-723 were on average of 4.1%, 49.9% and 65.2% in the three lung fluids, respectively, and (3) the bioaccessibility of Pb in the NIST 2710a was on average of 0.04%, 7.9% and 55% in the three fluids, respectively (Table 3). The much higher metal release rates in ALF than in Gamble’s solution and/or PBS are expected since ALF is a more aggressive solution (lower pH). This finding is supported by various studies reporting increased metal release in acidic environments [48,55,56,64,68,73,74]. Thus, the bioaccessibility values of most elements were critically dependent on SLF pH. For the three other elements (Co, Cr, and Sr), the sequence ALF > Gamble’s solution > PBS was not observed. A high variability of Co and Sr bioaccessibility was recorded between the three lung fluids and within each fluid (i.e., high standard deviations; Table 3). For Cr, the soluble fraction was very low whatever the lung fluid used (<10%; Table 3). All these results show that metal release could also be strongly influenced by the chemical composition and the physicochemical properties of the dissolution media, and the effective surface area of particles during exposure. Variation in chemical compositions could favor the mobilization or immobilization of metals from SRMs.

Gamble’s solution is made up of inorganic salts, carbonates, chlorides, and phosphates and has a neutral pH of ca. 7.3. ALF has a comparatively different salt solution and low pH of ca. 4.5. In these acidic conditions, it has been shown that metal-chloride complexes can be formed due to presence of HCl and can be easily solubilized [55]. Alternatively, the neutral pH in conjunction with the presence of carbonates in Gamble’s solution may favor chemical formation of insoluble complexes. PBS and Gamble’s solution have the same pH but induce significantly different metal bioaccessibility. PBS is considered as a “non-aggressive” solution and its relatively lower leaching efficiency was attributed to the absence of proteins and organic compounds that are represented by citrate, glycine and cysteine in Gamble’s solution. The organic compounds play a major role in the dissolution of metals from SRMs by acting as weak chelating agents, dissolving otherwise insoluble compounds and incorporating them into their amino acid structure. These results imply that the more complex composition of Gamble’s solution determines the release process.

Even if different bioaccessibilities of metals were evidenced for the three lung fluids tested and for the three SRMs studied (Table 3), some behaviors were observed: (1) the lowest bioaccessible percentages in PBS were noted for Ni, Pb and Ba (<1%), (2) the elements Cr and Ni were observed to have the lowest bioaccessibility in Gamble’s solution (<4%), while Cd, Cu and Zn had the highest, (3) Cr had also a low bioaccessibility in ALF (<9%), while the elements Cd, Cu, Pb, and Zn associated with all examined SRM were found to be the most bioaccessible in this lung fluid, (4) the other elements showed intermediate behavior with an intersample variability for the three SRMs.

The different percentages of metallic elements release can be attributed to differences in their mobilities, tendencies to form soluble complexes and transformations in the lung environment. The bioaccessibility is dependent on chemical status of metallic elements, which can be present in the synthetic fluids as mixed oxides, carbonates, aluminate, phosphate, silicate and mixed sulfides and chlorides. Metal oxides, chlorides, carbonates can be generally easily dissolved in lung fluids, while metal sulfides, phosphate and silicate are common insoluble compounds [75]. The presence of phosphate in PBS may limit Pb solubility [75,76], which could explain its low bioaccessible percentage. In the three synthetic lung fluids, the low bioaccessibility of Ni is due to the presence of insoluble compounds. In Gamble’s solution and ALF, the presence of citrate compounds could lead to Ni chelation [77]. Pyruvate and lactate, both present in ALF, are weak chelating agents and could lead to the dissolution of some Ni compounds [78], which could explain a slightly higher bioaccessibility in ALF than in Gamble’s solution. The results evidenced that elements such as Cd, known to be often in soluble-exchangeable forms (i.e., cadmium oxide and chloride), were highly bioaccessible, in particular in Gamble’s solution and ALF. The relatively high bioaccessibility of Cu and Zn in Gamble’s solution may be due to the presence of cysteine in this fluid. These elements have high affinity for thiol groups provided by cysteine as these complexes are easily dissolved [79,80].

The results of the extraction experiments for the three test SRMs (BCR-723, NIST 2710a and NIST 1648a) exposed to lung fluids showed that some metal releases changed with substrates. To resume, BCR-723 corresponds to a road dust material collected from the ceiling of a tunnel (with a median value of particle size of 14.6 µm), NIST 1648a was collected in an urban area (median value of 5.85 µm) and NIST 2710a corresponds to a highly contaminated agricultural soil (a particle size fraction of <74 µm, with no data about the median value). For example, the bioaccessible percentage of Sr determined by ALF was significantly lower at agricultural sites (2.3%) than those found in urban areas (56.2% and 50.9%, respectively, for BCR-723 and 1648a) (Table 3). A similar behavior was observed for Zn with 35.3% released in ALF from agricultural soil compared to 76.8% from road dust and 66.2% from urban areas (Table 3). In the case of Mn, the road dust sample (BCR-723) showed much lower percentage of release (5.5%) in ALF than either the urban area sample (1648a; 46.8%) or the agricultural soil sample (2710a; 44.3%). These differences in bioaccessibility were linked with some possible variations in speciation of these elements between samples [81,82], which can be attributed to different transformations for the metallic elements once they are deposited in the environment. Such transformations include complexation by inorganic and organic ligands leading to the formation of more or less soluble and mobile species. The high mobility could be due in part to the association of the element with smaller emitted particle size fractions. Other factors such as the elemental species of metals present in SRMs fractions may also explain the higher bioaccessibility of elements in lung fluids.

### 3.3. Influence of Addition of Surfactant in the Gamble’s Solution on Lung Bioaccessibility

To be in a more physiologically relevant environment with respect to the human respiratory tract, Gamble’s solution has been modified in many studies by adding organic acids, proteins, and surfactants [63,66,83]. These additional components were also expected to increase metal dissolution kinetics and hence their bioaccessibility. In the present study, the Gamble’s solution used was based on Broadway et al. [19], in which proteins and organic compounds are represented by citrate, glycine and cysteine (Table 2). A modified Gamble’s solution was tested, in which a surfactant (DPPC) was added (Table 2). Surfactant DPPC is a phospholipid secreted by type 2 cells and located in alveoli of lungs. This compound is the most abundant lung surfactant [84] and is mainly employed in the pharmaceutical sector for dissolution testing of inhaled products, particularly for analyzing poorly soluble drug substances [51,52,85,86,87]. These different studies demonstrated that the addition of DPPC to lung fluids increased the wettability of particles, improved contact between leaching solution and substances and prevented aggregation. Although most of these studies used a DPPC concentration of 200 mg·L^−1^, a concentration of 100 mg·L^−1^ is suitable for mimicking the airway’s fluid surfactant properties [56,85]. Indeed, in human bronchoalveolar lavage (BAL) fluid, the median concentration of phosphatidylcholine was found to be 0.0074 g·L^−1^ of BAL fluid. It has been showed that BAL fluid contained 1/13.5 of this constituent [56]. Consequently, the concentration of DPPC in a SLF based on Gamble’s solution can be estimated (i.e., 100 mg·L^−1^). This concentration was adopted in the present study as part of the modified Gamble’s solution (Table 2).

**Figure 2:**
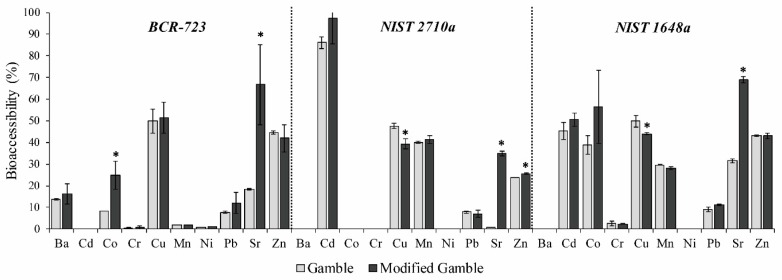
Bioaccessibility values (%; mean ± SD; *n* = 3) of metallic elements in the three reference materials for a S/L ratio of 1/5000 in Gamble’s solution and modified Gamble’s solution. * denotes significant differences (*p* < 0.05) between both Gamble’s solution and modified Gamble’s solution.

For each SRM studied, the percentages of bioaccessible metallic elements in Gamble’s solution and modified Gamble’s solution are shown in Figure 2. The results evidenced: (1) no significant difference between both solutions for Ba, Cd, Cr, Mn, Ni and Pb; (2) for the three SRMs, a higher bioaccessibility of Sr in modified Gamble’s solution (66.7%, 35.0% and 68.9%, respectively, for BCR-723, NIST 2710a and NIST 1648a) than in Gamble’s solution (18.5%, 0.8% and 31.5%, respectively, for BCR-723, NIST 2710a and NIST 1648a); (3) in Gamble’s solution, a Co bioaccessibility of 8.2% in BCR-723 and a Zn bioaccessibility of 23.7% in NIST 2710a, which were much lower than the 24.9% and 25.5% (respectively, for Co and Zn) in modified Gamble’s solution; and (4) a different behavior for Cu in NIST 2710a and 1648a, where a higher bioaccessibility was recorded in Gamble’s solution (respectively, 47.6% and 49.9%) than in modified Gamble’s solution (respectively, 39.4% and 44.0%). For Sr, the addition of DPPC showed a strong influence on its bioaccessibility in the three SRMs studied. The results for other metallic elements did not evidence the need and effectiveness of this surfactant.

## 4. Conclusions

The objective of this study was to assess bioaccessibility of metallic elements from reference materials for the inhalation route. The leaching solution (PBS, Gamble’s solution and ALF) composition and different solid/liquid ratios were tested for several elements (Ba, Cd, Co, Cr, Cu, Mn, Ni, Pb, Sr and Zn) on three standard reference materials. The results show that:

- Bioaccessibility values are critically dependent on SLF pH;
- Bioaccessibility is closely associated with the lung solution used. Indeed, the chemical composition of lung fluid (with similar pH) had an impact on pulmonary bioaccessibility and can favor the mobilization or immobilization of elements from SRMs;
- Addition of surfactant (DPPC) resulted in no significant change in the bioaccessibility of most of the metallic elements studied;
- Bioaccessibility was speciation- and element-dependent, with percentages varying from 0.04% for Pb to 86.0% for Cd;
- For PBS and Gamble’s solutions, S/L ratios influenced dissolution, while for ALF, bioaccessibility is independent of S/L ratios over a large range from 1/1000 to 1/10,000.

Because of the higher extraction of metallic elements obtained with the ALF, in which a relative stability of bioaccessibility was observed in a large range of S/L ratios from 1/1000 to 1/10,000, we suggest the use of this lung fluid. In further research, this protocol will be used to assess lung bioaccessibility of metals from smelter-impacted dusts.

## Figures and Tables

**Table 1:** Certified values (expressed in mg·kg^−1^) for selected metals in the three standard reference materials (SRMs) studied (BCR-723, NIST 2710a and NIST 1648a).

Elements	BCR-723	NIST 2710a	NIST 1648a
Ba	460 ± 33	792 ± 36	737 **^2^**
Cd	2.5 ± 0.4	12.3 ± 0.3	73.7 ± 2.3
Co	29.8 ± 1.6	5.99 ± 0.14	17.97 ± 0.68
Cr	440 ± 18	23 ± 6	402 ± 13
Cu	226 ± 3 **^1^**	3420 ± 50	610 ± 70
Mn	1280 ± 40	2140 ± 60	790 ± 44
Ni	171 ± 3	8 ± 1	81.1 ± 6.8
Pb	866 ± 16	5520 ± 30	6550 ± 330
Sr	254 ± 19	255 ± 7	215 ± 17
Zn	1660 ± 100	4180 ± 150	4800 ± 270

Data in brackets: Certified values not available for these elements in the reference material; **^1^** value from [62], **^2^** value from [65].

**Table 2:** Composition and properties of the different simulated lung fluids (SLFs) studied (PBS, Gamble’s solution, modified Gamble’s solution and ALF). PBS: phosphate-buffered saline; ALF: artificial lysosomal fluid.

Composition (g·L^−1^)	PBS ^1^	Gamble ^2^	Modified Gamble	ALF ^3^
NaCl	8.77	6.779	6.779	3.21
Na_2_HPO_4_	1.28			0.071
NaHCO_3_		2.268	2.268	
Trisodium citrate dihydrate		0.055	0.055	0.077
NH_4_Cl		0.535	0.535	
Glycine		0.375	0.375	0.059
NaH_2_PO_4_		1.872	1.872	
L-cysteine		0.121	0.121	
NaOH				6.0
Citric acid				20.8
CaCl_2_·2H_2_O		0.026	0.026	0.128
Na_2_SO_4_				0.039
MgCl_2_·6H_2_O				0.05
Disodium tartrate				0.09
Sodium lactate				0.085
Sodium pyruvate				0.172
KH_2_PO_4_	1.36			
DPPC **^4^** (surfactant)			0.01%	
**Properties**				
pH	7.3 ± 0.1	7.3 ± 0.1	7.3 ± 0.1	4.5 ± 0.1
Ionic strength (mol·L^−1^)	0.19	0.17	0.17	0.34

**^1^** [48]; **^2^** [19]; **^3^** [55]; **^4^** Dipalmitoylphosphatidylcholine.

**Table 3:** Bioaccessibility values (%; mean ± SD; *n* = 3) of metallic elements in the three reference materials for a S/L ratio of 1/5000 in the three lung fluids (PBS, Gamble’s solution and ALF).

Lung Fluids	Elements (%)									
	Ba	Cd	Co	Cr	Cu	Mn	Ni	Pb	Sr	Zn
BCR-723										
PBS	0.5 ± 0.3 **^a^**	<LD	14.0 ± 6.0 **^a^**	0.8 ± 0.5 **^a^**	4.1 ± 1.5 **^a^**	0.9 ± 0.0 **^a^**	<LD	<LD	22.1 ± 1.2 **^a^**	6.8 ± 0.8 **^a^**
Gamble	13.6 ± 0.4 **^b^**	<LD	8.2 ± 0.0 **^a^**	0.5 ± 0.3 **^a^**	49.9 ± 5.6 **^b^**	1.7 ± 0.0 **^b^**	0.8 ± 0.0 **^a^**	7.8 ± 0.6 **^a^**	18.5 ± 0.4 **^a^**	44.6 ± 0.8 **^b^**
ALF	35.7 ± 0.5 **^b^**	81.4 ± 7.6 **^a^**	39.8 ± 15.3 **^a^**	8.7 ± 0.0 **^b^**	65.2± 3.7 **^c^**	5.5 ± 0.1 **^c^**	24.1 ± 3.7 **^b^**	62.0 ± 3.2 **^b^**	56.2 ± 3.2 **^b^**	76.8 ± 2.2 **^c^**
NIST 2710a										
PBS	<LD	44.2 ± 21.2 **^a^**	95.1 ± 52.7 **^a^**	7.8 ± 0.0	8.3 ± 0.2 **^a^**	28.7 ± 0.4 **^a^**	<LD	0.04 ± 0.00 **^a^**	2.8 ± 0.0 **^a^**	6.2 ± 0.1 **^a^**
Gamble	<LD	86.0 ± 2.8 **^a^**	<LD	<LD	47.6 ± 1.4 **^b^**	40.1 ± 0.7 **^b^**	<LD	7.9 ± 0.4 **^b^**	0.8 ± 0.2 **^a^**	23.7 ± 0.1 **^b^**
ALF	25.3 ± 0.4 **^a^**	85.3 ± 8.4 **^a^**	35.1 ± 0.0 **^a^**	<LD	59.7 ± 1.4 **^c^**	44.3 ± 0.2 **^c^**	<LD	55.0 ± 0.5 **^c^**	2.3 ± 0.5 **^a^**	35.3 ± 0.1 **^c^**
NIST 1648a										
PBS	<LD	24.1 ± 6.2 **^a^**	3.3 ± 0.0 **^a^**	1.3 ± 0.4 **^a^**	7.3 ± 1.8 **^a^**	16.4 ± 1.4 **^a^**	<LD	<LD	29.0 ± 23.8 **^a^**	4.3 ± 0.2 **^a^**
Gamble	<LD	45.2 ± 4.0 **^b^**	38.8 ± 4.3 **^b^**	2.7 ± 1.0 **^a^**	49.9 ± 2.7 **^b^**	29.6 ± 0.2 **^b^**	3.3 ± 1.2 **^a^**	9.1 ± 0.9 **^a^**	31.5 ± 0.9 **^a^**	43.2 ± 0.2 **^b^**
ALF	52.8 ± 1.9 **^a^**	65.6 ± 5.5 **^c^**	35.0 ± 16.5 **^b^**	8.7 ± 0.9 **^b^**	55.0 ± 1.1 **^c^**	46.8 ± 2.6 **^c^**	12.2 ± 4.1 **^a^**	75.9 ± 2.2 **^b^**	50.9 ± 3.2 **^a^**	66.2 ± 2.3 **^c^**

LD: Limit of detection; Lowercase letters (a–c) denote significant differences (*p* < 0.05) between the three lung fluids for each element.

## Supplementary Materials

The following are available online at www.mdpi.com/1660-4601/14/2/112/s1, Table S1: Bioaccessibility values (%; mean ± SD; *n* = 3) of metallic elements in BCR-723 obtained through the four lung fluid extractions according to the four solid-to-liquid (S/L) ratios, Table S2: Bioaccessibility values (%; mean ± SD; *n* = 3) of metallic elements in NIST 2710a obtained through the four lung fluid extractions according to the four S/L ratios, Table S3: Bioaccessibility values (%; mean ± SD; *n* = 3) of metallic elements in NIST 1648a obtained through the four lung fluid extractions according to the four S/L ratios.
