# Supplementary material for: In Vitro Investigations of Human Bioaccessibility from Reference Materials Using Simulated Lung Fluids
Source: Int J Environ Res Public Health. 2017 Jan 24;14(2):112. doi: 10.3390/ijerph14020112 (PMC5334666; doi:10.3390/ijerph14020112)
Supplement: Supplementary file 1 [file ijerph-14-00112-s001.pdf]

Supplementary Materials: In Vitro Investigations of Human Bioaccessibility from Reference Materials Using Simulated Lung Fluids

Aurélie Pelfrêne, Mark R. Cave, Joanna Wragg and Francis Douay

Table S1. Bioaccessibility values (%; mean ± SD; *n* = 3) of metallic elements in BCR-723 obtained through the four lung fluid extractions according to the four solid-to-liquid (S/L) ratios.

S/L Ratios	Ba	Cd	Co	Cr	Cu	Mn	Ni	Pb	Sr	Zn
					PBS					
1/1000	0.6 ± 0.1	<LD	3.6 ± 3.5	0.4 ± 0.1	9.8 ± 0.8	0.1 ± 0.0	2.5 ± 0.5	<LD	9.0 ± 0.3	1.0 ± 0.0
1/2000	0.1 ± 0.0	<LD	8.2 ± 4.4	0.4 ± 0.0	8.9 ± 2.2	0.8 ± 0.0	1.5 ± 0.3	<LD	20.2 ± 1.2	2.1 ± 0.2
1/5000	0.5 ± 0.3	<LD	14.0 ± 6.0	0.8 ± 0.5	4.1 ± 1.5	0.9 ± 0.0	<LD	<LD	22.1 ± 1.2	6.8 ± 0.8
1/10,000	1.1 ± 0.5	<LD	16.0 ± 4.0	1.6 ± 1.4	1.0 ± 0.0	1.3 ± 0.1	<LD	<LD	24.2 ± 2.2	12.6 ± 0.6
					Gamble					
1/1000	<LD	<LD	9.8 ± 0.1	0.8 ± 0.1	49.3 ± 2.1	0.5 ± 0.1	6.2 ± 0.8	<LD	15.7 ± 0.8	2.7 ± 2.0
1/2000	8.4 ± 0.3	<LD	11.8 ± 1.8	0.8 ± 0.3	54.4 ± 3.4	1.3 ± 0.0	7.1 ± 0.4	1.7 ± 0.8	18.6 ± 0.4	30.8 ± 0.3
1/5000	13.6 ± 0.4	<LD	8.2 ± 0.0	0.5 ± 0.3	49.9 ± 5.6	1.7 ± 0.0	0.8 ± 0.0	7.8 ± 0.6	18.5 ± 0.4	44.6 ± 0.8
1/10,000	19.8 ± 6.3	<LD	<LD	0.5 ± 0.1	56.3 ± 4.9	2.8 ± 0.7	5.9 ± 0.0	12.3 ± 3.6	19.8 ± 0.1	57.7 ± 0.8
					Modified Gamble					
1/1000	1.0 ± 0.3	26.5 ± 1.9	16.0 ± 4.9	1.3 ± 0.8	51.5 ± 0.7	0.4 ± 0.1	6.7 ± 1.0	1.0 ± 0.5	28.5 ± 2.2	3.0 ± 1.7
1/2000	7.7 ± 3.5	17.1 ± 15.4	22.1 ± 3.9	1.2 ± 0.2	51.7 ± 5.4	1.2 ± 0.2	6.3 ± 0.8	3.8 ± 2.2	45.8 ± 1.7	27.2 ± 5.9
1/5000	16.2 ± 4.5	79.4 ± 14.4	24.9 ± 6.4	0.9 ± 0.8	51.4 ± 7.2	1.8 ± 0.1	1.1 ± 0.0	11.9 ± 4.8	66.7 ± 18.6	42.0 ± 6.3
1/10,000	21.9 ± 2.3	95.0 ± 0.1	29.6 ± 7.4	1.9 ± 0.7	63.0 ± 5.1	2.0 ± 0.0	<LD	22.1 ± 11.4	85.5 ± 3.6	53.9 ± 1.5
					ALF					
1/1000	42.0 ± 13.7	75.4 ± 10.2	52.0 ± 16.8	12.4 ± 4.6	71.1 ± 8.8	5.5 ± 0.2	34.8 ± 2.3	67.2 ± 1.8	50.7 ± 1.8	77.8 ± 1.8
1/2000	47.5 ± 19.8	77.4 ± 8.0	59.7 ± 27.2	12.7 ± 5.9	75.6 ± 4.3	5.6 ± 0.0	33.8 ± 0.5	66.4 ± 0.4	52.8 ± 0.3	79.2 ± 0.8
1/5000	35.7 ± 0.5	81.4 ± 7.6	39.8 ± 15.3	8.7 ± 0.0	65.2 ± 3.7	5.5 ± 0.1	24.1 ± 3.7	62.0 ± 3.2	56.2 ± 3.2	76.8 ± 2.2
1/10,000	39.0 ± 0.1	74.4 ± 3.0	51.8 ± 0.1	8.3 ± 0.1	75.8 ± 0.1	5.6 ± 0.0	47.3 ± 0.1	59.7 ± 0.1	63.5 ± 0.1	74.9 ± 0.1

LD: Limit of detection; PBS: phosphate-buffered saline; ALF: artificial lysosomal fluid.

Table S2. Bioaccessibility values (%; mean ± SD; *n* = 3) of metallic elements in NIST 2710a obtained through the four lung fluid extractions according to the four S/L ratios.

S/L Ratios	Ba	Cd	Co	Cr	Cu	Mn	Ni	Pb	Sr	Zn
PBS										
1/1000	<LD	28.9 ± 10.3	5.9 ± 1.7	<LD	3.4 ± 0.4	21.6 ± 3.0	<LD	<LD	0.2 ± 0.0	2.1 ± 0.3
1/2000	<LD	43.8 ± 6.4	27.5 ± 12.5	1.4 ± 1.0	5.9 ± 0.1	31.9 ± 1.2	<LD	0.2 ± 0.0	3.3 ± 0.1	4.1 ± 0.4
1/5000	<LD	44.2 ± 21.2	95.1 ± 52.7	7.8 ± 0.0	8.3 ± 0.2	28.7 ± 0.4	<LD	0.04 ± 0.00	2.8 ± 0.0	6.2 ± 0.1
1/10,000	<LD	69.7 ± 11.5	61.9 ± 0.1	5.5 ± 0.0	11.7 ± 3.6	33.1 ± 8.8	<LD	0.1 ± 0.1	<LD	8.6 ± 2.0
Gamble										
1/1000	<LD	56.8 ± 9.0	<LD	<LD	55.3 ± 4.2	33.0 ± 2.3	<LD	2.3 ± 0.3	1.3 ± 0.6	13.7 ± 2.2
1/2000	<LD	61.9 ± 10.4	<LD	<LD	52.1 ± 3.0	37.3 ± 1.8	<LD	3.7 ± 0.2	0.8 ± 0.0	18.0 ± 0.9
1/5000	<LD	86.0 ± 2.8	<LD	<LD	47.6 ± 1.4	40.1 ± 0.7	<LD	7.9 ± 0.4	0.8 ± 0.2	23.7 ± 0.1
1/10,000	<LD	82.7 ± 16.1	<LD	<LD	62.4 ± 1.4	56.9 ± 12.5	<LD	12.5 ± 0.6	1.4 ± 0.1	34.0 ± 6.8
Modified Gamble										
1/1000	0.1 ± 0.0	76.1 ± 2.4	22.9 ± 23.4	<LD	49.4 ± 2.9	31.8 ± 1.7	<LD	2.2 ± 0.1	7.0 ± 0.3	13.2 ± 1.0
1/2000	0.3 ± 0.1	77.3 ± 0.1	32.5 ± 0.1	<LD	47.4 ± 2.0	36.6 ± 1.6	<LD	3.8 ± 0.1	13.9 ± 1.4	18.6 ± 0.4
1/5000	1.0 ± 0.0	97.2 ± 11.7	47.5 ± 0.1	<LD	39.4 ± 2.2	41.3 ± 2.0	<LD	7.1 ± 1.5	35.0 ± 1.1	25.5 ± 0.4
1/10,000	1.4 ± 2.0	88.1 ± 0.1	55.6 ± 25.8	<LD	36.9 ± 4.9	40.9 ± 2.2	<LD	11.8 ± 1.1	46.7 ± 5.8	25.9 ± 4.2
ALF										
1/1000	23.2 ± 0.4	94.4 ± 5.1	22.2 ± 9.3	<LD	62.0 ± 0.6	43.4 ± 1.6	<LD	51.4 ± 1.1	2.9 ± 0.3	36.7 ± 1.6
1/2000	24.9 ± 0.9	93.5 ± 5.8	32.3 ± 22.9	<LD	61.2 ± 1.8	43.1 ± 1.2	<LD	55.0 ± 1.4	2.6 ± 0.5	36.1 ± 0.7
1/5000	25.3 ± 0.4	85.3 ± 8.4	35.1 ± 0.0	<LD	59.7 ± 1.4	44.3 ± 0.2	<LD	55.0 ± 0.5	2.3 ± 0.5	35.3 ± 0.1
1/10,000	26.8 ± 0.0	89.4 ± 0.1	36.5 ± 0.1	<LD	61.8 ± 0.3	45.7 ± 0.1	<LD	56.1 ± 0.1	3.1 ± 0.1	35.9 ± 0.1

Table S3. Bioaccessibility values (%; mean ± SD; *n* = 3) of metallic elements in NIST 1648a obtained through the four lung fluid extractions according to the four S/L ratios.

S/L Ratios	Ba	Cd	Co	Cr	Cu	Mn	Ni	Pb	Sr	Zn
PBS										
1/1000	<LD	9.1 ± 0.5	9.5 ± 3.7	0.4 ± 0.1	9.6 ± 0.4	7.4 ± 0.3	6.6 ± 1.8	<LD	14.1 ± 0.5	0.7 ± 0.0
1/2000	<LD	15.4 ± 1.5	8.2 ± 0.9	0.6 ± 0.3	11.4 ± 1.0	13.5 ± 0.5	5.6 ± 2.6	<LD	16.5 ± 0.6	2.0 ± 0.1
1/5000	<LD	24.1 ± 6.2	3.3 ± 0.0	1.3 ± 0.4	7.3 ± 1.8	16.4 ± 1.4	<LD	<LD	29.0 ± 23.8	4.3 ± 0.2
1/10,000	<LD	29.6 ± 1.2	8.9 ± 0.1	1.8 ± 0.3	8.4 ± 4.5	23.0 ± 0.9	<LD	<LD	32.1 ± 2.2	7.1 ± 0.1
Gamble										
1/1000	<LD	11.0 ± 2.0	16.5 ± 5.9	1.4 ± 0.4	52.0 ± 13.2	8.5 ± 2.8	26.7 ± 8.8	0.4 ± 0.1	15.8 ± 0.7	4.3 ± 1.3
1/2000	<LD	34.2 ± 7.5	17.4 ± 4.0	1.8 ± 0.5	51.9 ± 6.1	23.4 ± 2.4	18.9 ± 0.9	2.6 ± 0.1	32.0 ± 9.2	25.4 ± 0.4
1/5000	<LD	45.2 ± 4.0	38.8 ± 4.3	2.7 ± 1.0	49.9 ± 2.7	29.6 ± 0.2	3.3 ± 1.2	9.1 ± 0.9	31.5 ± 0.9	43.2 ± 0.2
1/10,000	<LD	49.7 ± 2.1	56.7 ± 2.0	3.7 ± 0.0	45.7 ± 11.6	29.4 ± 5.4	<LD	16.3 ± 0.4	37.4 ± 0.1	49.2 ± 6.9
Modified Gamble										
1/1000	0.1 ± 0.0	15.2 ± 0.4	26.7 ± 5.4	1.4 ± 0.1	46.0 ± 2.7	12.2 ± 0.6	22.2 ± 2.1	0.9 ± 0.1	28.2 ± 2.5	7.0 ± 0.5
1/2000	0.4 ± 0.1	33.2 ± 1.8	34.9 ± 11.9	1.9 ± 0.5	48.4 ± 1.7	22.4 ± 0.6	18.2 ± 3.6	3.1 ± 0.0	40.9 ± 1.7	27.3 ± 0.4
1/5000	2.4 ± 0.5	50.7 ± 3.1	56.5 ± 6.9	2.4 ± 0.2	44.0 ± 0.4	28.1 ± 0.8	<LD	11.2 ± 0.5	68.9 ± 1.4	43.0 ± 1.2
1/10,000	2.3 ± 0.3	64.9 ± 16.0	74.8 ± 8.5	2.5 ± 0.9	53.3 ± 4.7	33.4 ± 1.4	<LD	16.3 ± 3.9	41.9 ± 1.2	55.3 ± 3.5
ALF										
1/1000	38.9 ± 1.2	69.9 ± 2.0	33.9 ± 2.1	8.9 ± 0.5	57.5 ± 1.6	45.9 ± 0.6	30.9 ± 1.8	74.0 ± 2.8	51.0 ± 3.6	67.4 ± 2.1
1/2000	46.7 ± 1.8	68.4 ± 3.8	40.6 ± 9.6	8.5 ± 0.6	52.6 ± 3.7	45.2 ± 2.5	25.2 ± 5.7	72.3 ± 2.6	49.9 ± 4.1	65.7 ± 2.8
1/5000	52.8 ± 1.9	65.6 ± 5.5	35.0 ± 16.5	8.7 ± 0.9	55.0 ± 1.1	46.8 ± 2.6	12.2 ± 4.1	75.9 ± 2.2	50.9 ± 3.2	66.2 ± 2.3
1/10,000	52.7 ± 1.9	72.2 ± 6.4	52.9 ± 45.1	7.0 ± 0.9	52.7 ± 3.1	46.1 ± 1.3	<LD	76.0 ± 2.3	52.8 ± 2.7	66.1 ± 1.9

© 2017 by the authors; licensee MDPI, Basel, Switzerland. This article is an open access article distributed under the terms and conditions of the Creative Commons by Attribution (CC-BY) license (http://creativecommons.org/licenses/by/4.0/).
